# Evaluating an Integrative Theoretical Framework for HIV Sexual Risk among Juvenile Justice involved Adolescents

**Published:** 2013-06-23

**Authors:** Renee E Magnan, Tiffany J Callahan, Benjamin O Ladd, Eric D Claus, Kent E Hutchison, Angela D Bryan

**Affiliations:** 1 Department of Psychology, Washington State University Vancouver, USA; 2 University of Colorado Boulder, USA; 3 Center on Alcoholism, Substance Abuse and Addictions, USA; 4 Mind Research Network, USA

**Keywords:** Neurocognition, Genetics, Justice-involved adolescents, Risky sex, Alcohol, Condom

## Abstract

Juvenile justice involved youth are at great risk for negative outcomes of risky sexual behavior including HIV/AIDS. Given the strong connection between alcohol use and risky sex in this population, it is important to consider alcohol use in interventions designed to decrease risky sexual behavior. This paper provides support for an integrative translational model that incorporates psychosocial, neurobiological, and genetic factors to better predict alcohol-related sexual risk behavior. Specifically, we present the design, methods, and baseline data from a complex randomized control trial, Project SHARP (Sexual Health and Adolescent Risk Prevention) in order to illustrate how this broad array of factors can best predict alcohol-related sexual risk behavior. Participants were justice-involved adolescents (n=284) who completed an fMRI and self-report assessments prior to randomization to either a sexual risk plus alcohol risk reduction group intervention or to an information-only contact control group intervention. Structural equation modeling was utilized and findings supported the hypothesized relationships in the translational model. Preliminary data suggest that interventions among justice-involved adolescents targeting alcohol-related sexual risk behavior may be more effective if a biopsychosocial approach is considered.

## Introduction

Adolescence is a unique developmental period of exploration and risk. Individuals begin taking responsibility for their decisions and behavior in the context of more adult activities while the precise regions of the brain most able to weigh consequences and self-regulate in tempting situations are still developing [1]. Thus, adolescents are poorly neurocognitively equipped for this exploration. Unfortunately, many of the behaviors “typical” to adolescent exploration can have serious negative consequences [2].

One of the most common adolescent exploratory behaviors that can lead to unintended negative consequences is unprotected sex. Youth under age 25 are at risk for sexually transmitted illnesses (STIs) including the human immunodeficiency virus (HIV) [3]. Indeed, 50% of all new STI/HIV infections worldwide occur between the ages of 15 and 24 [4]. Youth involved with the justice system are at particularly high risk for negative outcomes as a result of risky sexual behavior [5]. In comparison to the general adolescent population, justice-involved adolescents are younger at first intercourse, have more sex partners, and lower rates of condom use [5]. These same young people remain at continued risk of HIV into adulthood [6].

There is a strong relationship between sexual risk behavior and substance use, particularly among high risk adolescents [7,8]. Alcohol is commonly used among adolescents [9] and alcohol-use disorders are more prevalent among justice-involved youth than their non-justice-involved peers [10]. Given the strong connection between alcohol use and risky sexual behavior, interventions to decrease risky sexual behavior that *include alcohol use* among adolescents are critically important. While some current intervention approaches show promise [11,12], they often have small effect sizes, decay over time, or do not work universally well for all adolescents [13]. One key to developing more effective interventions may be to develop a clearer understanding of the variables that influence risky sex in this population. The vast majority of work focuses on psychosocial factors (e.g., motivations) that influence condom use [14,15], with little effort focused on the biological underpinnings of risk behavior specifically neurocognitive and genetic factors.

### Neurocognitive structure and function

The neurocognitive components of risk-taking behavior include the inability to inhibit a prepotent response, problems with delay of gratification, and risky decision-making. These processes are intimately related to the execution of self-regulation [16]. The neurological substrates involved in such decisions include the ventral medial prefrontal cortex (vmPFC) [17], the anterior cingulate cortex (ACC) [17,18] the inferior frontal gyrus [19,20] and the orbitofrontal cortex (OFC) [21,22]. These regions have received support as being associated with risky decision making through imaging studies linking activation to performance on cognitive tasks including a delay discounting task [23], and the Balloon Analogue Risk Task (BART) [24,25].

Such variation in neurocognitive function may be important to consider when implementing behavioral interventions meant to decrease risk-taking. Assessments of processes such as delay discounting and impulsivity “may be able to predict who is at risk [for problem behaviors] and who is most likely to benefit from interventions” [26]. However, there is no published evidence that such measures moderate the effectiveness of interventions to reduce alcohol-related sexual risk behavior (or any other risk behavior for that matter).

### Genetic factors

Given the importance of brain-based endophenotypes related to impulsivity and risk-taking, genes associated with these constructs may underlie variation in brain function during tasks that tap these characteristics. Because there are hundreds if not thousands of genes implicated in variation in neurocognitive function related to risk-taking, we selected candidate genes with support in the literature. A variable number tandem repeat (VNTR) in thedopamine receptor D4 (DRD4) gene, as well as other genes that influence dopamine function (DRD2, DAT1, COMT), may be associated with activity in the ventral striatum during a task that taps preference for immediate over delayed rewards [27]. Recent work on the cholinergic muscarinic receptor 2 (CHRM2) gene suggests that its variants are associated with novelty-seeking and sensation-seeking [28]; and with measures of disinhibition [29]. The cannabinoid receptor 1 (CNR1) gene is associated with both alcohol cue-elicited neurocognitive activation [30] and alcohol-related behavior [31]. Examining genetic factors associated with risk behavior allows for the development of a theoretical framework that mechanistically links genetic variation to neurocognitive function and behavior.

### Integrative translational approach

The existing evidence supports an integrative translational approach incorporating neurocognitive and genetic factors into a biopsychosocial framework for the prediction of alcohol-related risky sexual behavior. The purpose of this paper is to present the design, methods, and baseline data from the SHARP (Sexual Health and Adolescent Risk Prevention) intervention trial to illustrate how neurocognitive and genetic variables can be integrated into a biopsychosocial framework to better predict alcohol-related sexual behavior. Specifically, we will describe the study and rationale of Project SHARP; describe the sample; and provide a preliminary test of the integrative translational model guiding our approach. The goal of the broader study was to test moderators of the efficacy of a sexual risk reduction intervention that included alcohol use content utilizing an intervention that has been previously successful [11].

## Methods

Project SHARP was a 12-month randomized controlled trial conducted at the University of New Mexico (UNM) and Mind Research Network (MRN) in Albuquerque, NM. Participants completed a baseline session (fMRI scan, DNA collection, and self-report assessment) and were randomized to receive either a group-based sexual risk plus alcohol risk reduction intervention which included a motivational enhancement therapy (GPI+GMET) or a group-based information-only contact control (GINFO). Follow-up assessments were completed at 3, 6, 9, and 12 months post-intervention. Participants were compensated up to $185 for completing the study. UNM’s IRB and the national Office of Human Research Protections approved this project. A certificate of confidentiality was also obtained from NIH/NIAAA.

### Participants

Participants were in a court ordered diversion program which provides daily supervision to help youth reintegrate into their school environments, but youth live in the community and are not in custody. Participants were recruited via research assistants (RAs) visiting the program site and announcing the opportunity for participation. Adolescents who expressed interest in the study met individually with a RA to complete informed assent/consent and undergo screening for inclusion/exclusion. Once assent was obtained, parent/guardian consent was obtained via telephone consistent with our prior work [32].

Participants had to be between the ages of 14–18, be able to read and speak English, give informed assent (or informed consent for 18 year olds), have verbal consent from a parent/legal guardian, not be taking psychotropoic medications, and be willing to accept random assignment. Individuals were excluded from the MRI (but not from the intervention and follow-up components) if they had a history of injury to the brain or a brain-related medical condition, non-removable metallic implants, received a tattoo less than two weeks prior to the scan, or were pregnant.

**Figure 1:**
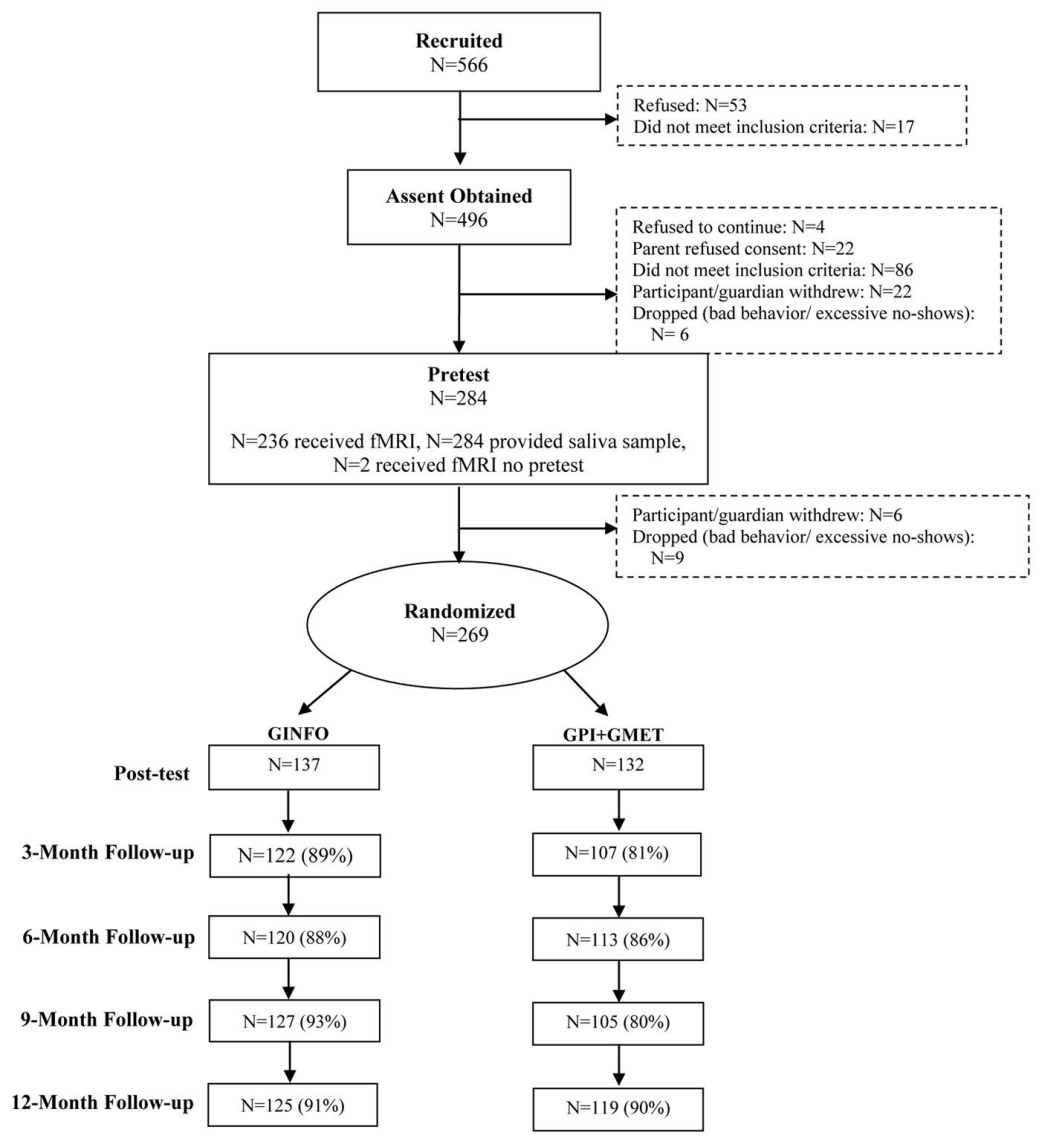
Project SHARP Retention Rates. *Note.* Any participant lost to follow-up was due to not being able to contact the participant.

Five hundred and sixty-six adolescents were recruited; 282 were dropped, withdrew, or the parent/guardian refused consent. A total of 284 individuals (205 male; 79 female) completed the baseline assessments; 269 were randomly assigned to an intervention condition (GINFO=137, GPI+GMET=132). We compared the two intervention groups on baseline characteristics (e.g., gender, age, individual-difference traits, alcohol and sexual risk behavior, motivation for condom use). Employing a bonferroni correction for alpha inflation (*p*<.001), no group differences were found across 45 comparisons suggesting randomization was successful (all comparison *p*s>.10). Retention rates were 85.1%, 86.6%, 86.2%, and 90.7% at the 3-, 6-, 9-, and 12-month follow-ups, respectively (see Figure 1).

### Intervention procedure

After completing baseline assessments, participants were scheduled for their single-session group interventions conducted with a trained (at least bachelor’s level) intervention leader.

*GINFO* was designed to increase participants’ knowledge about HIV/AIDS and other STIs. This single one-hour session provided information on the symptoms, consequences, and methods for the prevention of STIs. GINFO included a didactic presentation on how one might acquire various STIs (with an emphasis on HIV), an informational video on the signs/symptoms of different STIs and safer-sex strategies, and a question-and-answer segment.

The theory-based *GPI+GMET* was designed to target motivational constructs from the Theory of Planned Behavior (TPB) [33] previously established to be associated with condom use in adolescents [14,15] in a non-confrontational manner that supports the autonomy of participants while encouraging risk reduction. GPI+GMET included discussion of alcohol-related topics designed to enhance the implementation of skills covered in the intervention and encourage problem solving around high-risk situations. This intervention included five sections with each section building on the previous section. *Section 1* covered general information on HIV/STI transmission. *Section II* addressed motivation to engage in safer-sex behavior and activities designed to increase condom use self-efficacy*. Section III* included a discussion of reasons to use condoms and how one might do so, and targeted problem-solving strategies for high risk situations. *Section IV* focused on scenarios where adolescents are likely to disregard safer-sex practices, particularly when alcohol is involved. This section included a group alcohol risk reduction MET component (GMET) [34]. *Section V* included exercises designed to consolidate plans for behavior change. Table 1 provides the general organization of GPI+GMET.

Our research team aims to develop, test, and determine the efficacy for an intervention that can be implemented in the detention setting. The ultimate goal is to provide such facilities with the necessary materials and training to conduct the interventions in the future. If such interventions are to be integrated within these detention programs, it is necessary to use a format and length that can be implemented within the confines of a short-term detention setting. Thus, we utilized a single-session intervention approach. Single-session group interventions have been shown to result in meaningful behavior change [35] including the intervention used in the current study [11]. Specifically, in our prior work, we showed that the GPI+GMET intervention resulted in greater condom use 12-months post intervention compared to a control condition. Additionally, in a meta-analytic review of HIV prevention interventions for adolescents, Johnson et al. [35] concluded that the content of interventions (i.e., emphasizing condom-use skills and motivation to use condoms) may be more important for changing condom use rather than the number of sessions in the intervention.

### Self-Report assessments

Assessments completed at baseline, 3-, 6-, 9-, and 12-months post intervention measured TPB motivational constructs, sexual behavior, and alcohol use. Behavioral data were collected using a 30-day Time Line Follow-Back (TLFB) [36] interview procedure. Broader past history (lifetime and last three months) of sexual and alcohol use behavior was assessed using Audio Computer-Assisted Self-Interviewing technology on individual laptop computers. Using MediaLab [37], survey questions were displayed on a laptop computer screen and participants’ responses were digitally recorded. Summary scores were computed and higher scores in all cases indicate higher levels of a construct.

#### Sexual behavior and condom use

Following previous work [14,15], participants who reported having had sexual intercourse indicated the age of first intercourse, number of sexual partners (lifetime), frequency of intercourse in their lifetimes (1=A few times a year; 6=almost every day) and in the past three months (1=never; 6=almost every day), if they had ever been or gotten someone pregnant, and if they ever had an STI. Participants also indicated frequency of condom use and frequency of alcohol use during sex (both assessed during lifetime and past three months, 1=never; 5=always).

#### Alcohol use

Alcohol use was measured using a variation of the measure used by White and Labouvie [38]. Participants who indicated having had at least one full alcoholic drink indicated the age at which they started drinking, frequency of use over the last three months (1=never; 9=everyday), amount they typically drank at one time in the last three months (1=none; 10=more than 20), and how often they were drunk when drinking in the past three months (1=never; 5=always). An alcohol composite score was created by summing the scores of these three items, α = 0.78. *Norms for alcohol use* was assessed with three items. Participants indicated how often most of their friends drank alcohol and how often their friends got drunk when drinking alcohol (1=never; 5=always) and how much they agreed that most people their age drink to get drunk (1=disagree a lot; 4=agree a lot).

Alcohol Dependence over the past six months was assessed using the 10-item Alcohol Use Disorder Identification Test (AUDIT) [39], which has shown high reliability in adolescents generally [40] and in the current sample (α=0.83). The 23-item Rutgers Alcohol Problem Index (RAPI) [38] was specifically developed to evaluate *alcohol-related problems in adolescents*. Participants indicated how much a particular event had happened over the past six months as a result of their drinking (0=never, 1=1–2 times, 2=3–5 times, 3=6–10 times, 4=more than 10 times; α=0.94).

#### Individual difference measures

We assessed a range of individual differences related to risk behavior generally and to justice-involved adolescents specifically. We did not incorporate these individual-difference measures into the test of the translational model, but present them to provide a complete picture of the measures included in this study (see Table 2). *Impulsivity* was assessed with the Impulsive Sensation-Seeking Scale (IMPSS) [41]. Participants indicated whether each of 19 statements were true (0) or false (1) (e.g., “I’ll try anything once;” α = 0.76). *Externalizing behavior* was assessed with the 32-item Youth Self Report (YSR) [42]. Participants indicated if each statement (e.g., “I don’t feel guilty after doing something I shouldn’t”)was 0=not true, 1=somewhat/sometimes true, or 2=very/often true; α=0.93. *Depression* was assessed with the 10-item Child Depression Inventory (CDI) [43]. Individuals select one of three statements for each item that best describes how they felt over the past two weeks (e.g., 0=I am sad once in a while, 1=I am sad many times, 2=I am sad all the time), α=0.80. *Attention Deficit/Hyperactivity Disorder (AD/HD) symptoms* were assessed with the Conner’s-Wells Self-Report Scale-Short Version (CASS-S) [44]. On a 4-point scale (0=not at all true to 3=very much true), participants indicated how much they endorsed each statement (e.g., “I have too much energy to sit still for long,” α=0.90).

#### Motivation for safer sexual behavior

The TPB measures have been used in our prior work with justice-involved adolescents [15] and target attitudes and beliefs about condoms in general and in the context of alcohol use. Except where indicated, the range of these scales was 1–4 (“Disagree a lot” to “Agree a lot” or “Will not happen” to “Will definitely happen”).

*Attitudes toward condoms* was measured in two ways, a 13-item affective attitude measure (α=0.76; e.g., “Condoms can ruin the sexual mood” reversed), and a 5-item measure of global condom attitudes (α=.69; e.g., “Using a condom every time I have sex in the next three months would be…”). Response options were made on 7-point bipolar adjective scales (e.g., bad-good; unpleasant-pleasant). *Norms for condom use* was assessed with 8 items (α=0.93; e.g., “Most of my friends use condoms when they have sex”). *Self-efficacy for condom use* was assessed using the Condom Use Self-Efficacy Scale (CUSES) [45],α=0.83. Safer *sex intentions* were assessed with 13 items (e.g., “How likely is it that you will drink less/monitor your drinking the next time you are in a situation where you might have sex?” α= 0.88).

### Neurocognitive assessments

#### MRI data acquisition

MRI data were collected using a 3T Siemens Trio system equipped with a 12-channel receiver head phased array coil. Structural *MRI.* High resolution T1-weighted scans were collected with a multi-echo MPRAGE sequence (TR/TE/TI=2300/2.74/900ms, flip angle=8°, FOV=256 × 256mm, Slab thickness=176 mm, Matrix=256 × 256 × 176, Voxel size=1×1×1 mm, Number of echos=4, Pixel bandwidth=650 Hz, Total scan time=6 min). *Diffusion Tensor Imaging* (DTI). White matter was assessed with a DTI protocol sensitive to 35 diffusion directions. A single-shot EPI sequence was acquired with the following parameters: TR/TE=9100/86ms, FOV=256×256 mm, matrix size=64×64, slices=72, voxel size= 2.0×2.0×2.0mm^3^; total scan time=6:30 min.

Several functional MRI (fMRI) scans were collected on each participant. All of the following resting and task-based scans were collected using single-shot full k-space echo-planar imaging (EPI) with ramp sampling correction (TR/TE=2000/29ms, flip angle=75°, matrix size=64×64, field of view=240×240mm, slices=32, voxel size=3.8×3.8×3.5mm^3^). To improve the signal dropout and warping in the OFC, a tilting acquisition was applied [46]. All fMRI tasks were presented using Presentation or E-prime with a rear projection mirror system and responses were recorded using a custom fiber optic response pad. The initial start of each run was synchronized with a trigger pulse from the magnet in order to ensure precise temporal integration of stimulus presentation and fMRI data acquisition. Blood oxygen level dependent (BOLD) activation during specific contrasts defined by critical trial types in each task serves as the outcome measure of interest.

#### Functional connectivity MRI *(fcMRI)*

Resting-state fMRI sequences for purposes of intrinsic functional connectivity analyses were collected while participants viewed a fixation cross in the center of the screen. The total scan time for the task was 5:16 minutes.

We utilized two standard fMRI tasks. The *Go/No Go (Go1) Task* allows for the assessment of response inhibition and error monitoring while participants attempt to inhibit a prepotent response (see details in Stevens et al. [47]). The Delay Discounting *(DD)* task assesses the ability to delay gratification by examining choices between small, immediate rewards (e.g. $30 today) and larger, delayed rewards (e.g. $100 in a month) [48] (see details in Claus et al. [23]). We also included the relatively novel Balloon Analog Response Task *(BART)*, which we include in our translational model. The BART is an assessment of risk-taking [25] which has been associated with multiple forms of self-reported risk behavior [24]. The variant of the BART used in the current study used three balloons of different colors: blue, pink, and white. Blue and pink balloons exploded after 5 and 8 pumps on average, respectively, and white balloons did not explode but instead were used as control balloons [24]. For each balloon, a pseudo randomized predetermined number of pumps was used as the threshold for exploding: a minimum of two pumps for blue and five pumps for pink. The maximum number of pumps was 8 and 11. If the number of pumps exceeded the explosion threshold, the balloon expanded for 50msec, exploded (with explosion sound), and participants lost all the points they could have earned on that balloon. At any time, participants could cash out instead of continuing to pump. If participants cashed out, the balloon remained on the screen, and participants heard the sound of coins falling into a bank while the total number of points earned increased. Participants completed as many balloons as possible over the course of a 10-minute run. Key contrasts examined the differences between the pump responses for color balloons vs. white balloons.

### DNA collection, extraction, and storage

At baseline, participants delivered 5 ml of saliva into a sterile 50 ml conical centrifuge tube. The saliva sample was placed in a refrigerator and lysis buffer was added within 24 hours. Tris-HCl, pH 8; EDTA, pH 8; SDS and NaCl were added at 100 mM, 20 mM, 0.5% and 125 mM final concentrations, respectively. The tubes were refrigerated until the DNA was extracted within 48 hours or samples were frozen (for no more than six months) at −20C until thawed for completing the DNA prep. Proteinase K (0.2 mg/ml) was added and the tubes were incubated at 65°C for one hour to overnight.

Proteins and other contaminants were precipitated using 2mL of Qiagen protein precipitation solution followed by a 10-minute incubation on ice and 10-minute centrifugation at 3000xg. The supernatant was added to an equal volume of isopropyl alcohol and mixed gently. DNA sat at room temperature for 10 minutes to fully precipitate. The DNA was collected by centrifugation at 3000xg for 10 minutes. The DNA pellet was then rinsed once with 4 ml of 70% ethanol, collected by centrifugation at 3000 xg for five minutes. Ethanol was carefully poured out without losing the DNA pellet, placed inverted on the bench and allowed to air dry for 30 minutes. Once dry, the DNA was re-suspended in 500–1000 ul of TE buffer, allowed to sit 24–48 hours at room temperature and quantified via PicoGreen^®^ and fluorimetry (Qbit^®^, Life Technologies).

### Statistical analysis

**Figure 2:**
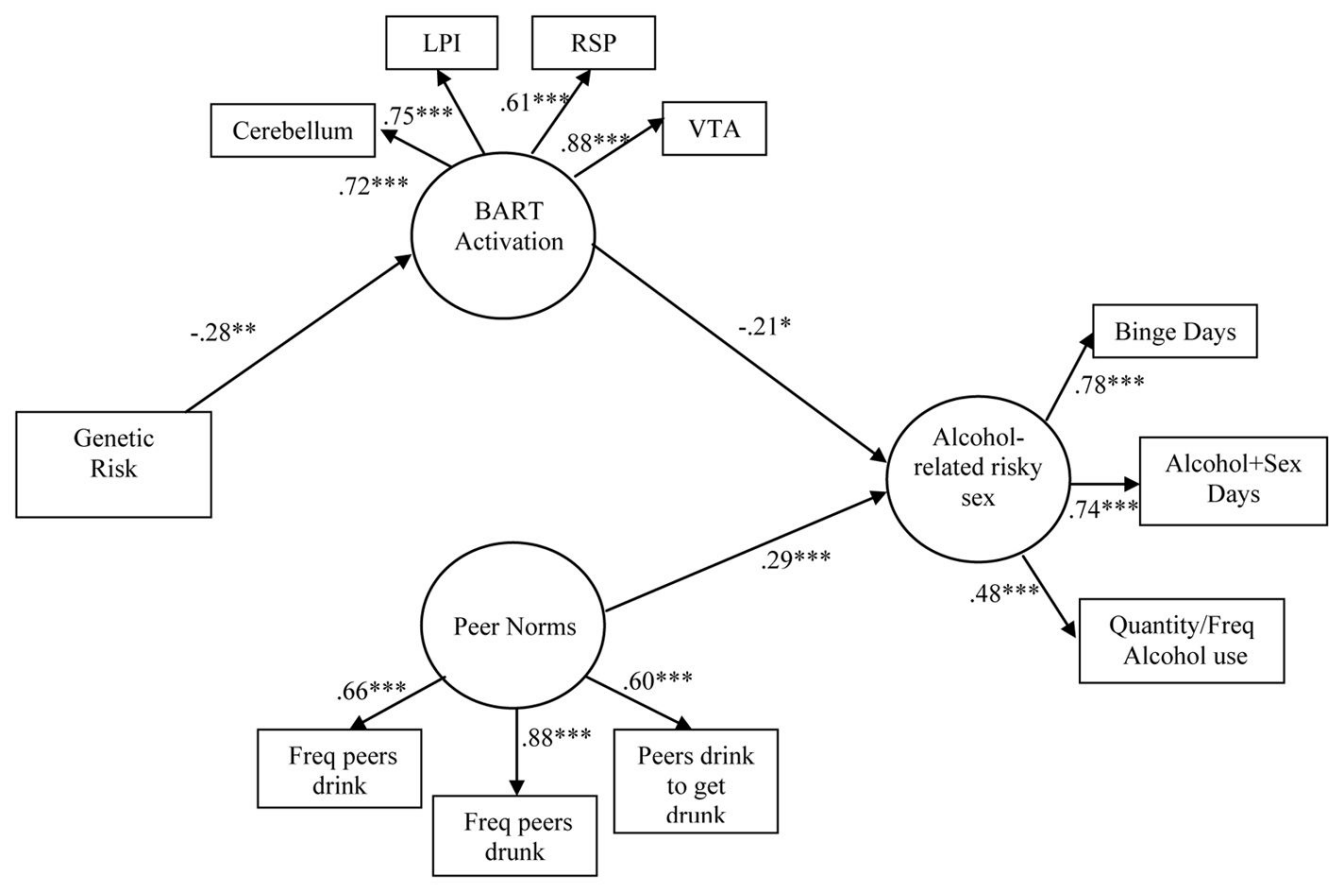
Integrative Model of Alcohol-Related Risky Sexual Behavior. *Note.* *p<.05, **p<.01, ***p<.001.

Prior to analysis, all variables were checked for normality. Because analyses were conducted on *a priori* hypothesized relationships, we maintained a significance level of *p*<0.05 [49]. Using the data from the BART, we constructed and tested a structural equation model including genetic factors, and estimated latent variables for neurocognitive components or risk-taking behavior, psychosocial motivational factors, and alcohol-related risky sex (see Figure 2).

## Results

### Demographics and baseline levels of risky behavior

Table 2 displays the baseline characteristics for all participants by gender. Participants were predominantly male (72.2%), Hispanic (63.0%), and on average were 16.16 years of age (SD=1.09). A large majority of participants reported ever having sex (80.1%), with an average mean number of 6.24 sexual partners (*SD*=6.52), and becoming sexually active at 13.12 years of age (SD=2.04) on average. Only 14.5% reported consistent condom use in their lifetimes. Most participants also reported ever drinking (90.5%) and they began drinking at 12.57 years of age (SD=2.28) on average. Nearly a third of those who drank and were sexually active admitted to using alcohol during intercourse (29.4%).

In general, relationships among baseline levels of risky behavior were significantly correlated such that greater frequency of sexual risk was associated with greater alcohol use. TPB motivational constructs were generally positively correlated with safer sexual behaviors (See Supplemental Table 1).

### Neurocognitve factors and risk-taking

There was a large amount of neurocognitive data collected in this study; far too much to adequately detail the findings for each domain of neurocognitive structure and function. Here, we present findings on BOLD activation during the BART, as an example of our approach to these data. We utilized activation parameters in key regions [cerebellum, left posterior insula (LPI), right superior parietal (RSP) and the ventral tegmental area (VTA)] for the contrast that compared activation during a risky decision to activation during a safe decision. These regions have been associated with risky decision-making in previous neurocognitive work [50–53].

### Association of genetic factors and neurocognitive function

Given the criticisms of single candidate gene approaches [54] we utilized the strategy of computing a linear combination of genes into a single genetic risk index [55]. We coded genotypes for the CHRM2 SNP (rs1455858), the CNR1 SNP (rs806380), and the DRD4 VNTR polymorphism such that higher values were related to higher risk (e.g., for the DRD4, individuals with two copies of the “short” variant were coded 0, while those with at least one copy of the “long” variant typically associated with higher risk behavior were coded 1). Thus, higher numbers on this index were associated with having more risk variants.

### Preliminary test of a translational model

One of the overarching goals of this study was to test linkages between genetic factors, neurocognitive factors, and behavior, and to understand whether these biologically based variables will account for variability in behavior over and above known psychosocial variables. Ninety-one participants were excluded from these analyses due to excessive movement or activation parameters 3 or more standard deviations away from the mean – the final sample for model testing included N=172. It is not feasible to include all possible variables into a single model, so we selected alcohol use and related sexual risk as the outcome variables of interest, given previously demonstrated associations between the BART and alcohol use in other work [24]. We estimated the model in Figure 2 in EQS (v.6.1). The genetic risk variable served as one exogenous variable. Given the importance of peer norms in adolescent risk behavior [56], we developed a latent variable using the three peer alcohol use norms items. The latent variable for neurocognitive function was comprised of BOLD activation during the BART in the cerebellum, LPI, RSP, and VTA. The behavioral outcome variable was comprised of number of binge drinking days in the past 30 days, number of days of intercourse concurrent with alcohol use in the past 30 days (both from the TLFB), and the alcohol composite score reflecting quantity and frequency of alcohol use in the past three months.

Due to scattered missing data for some participants, we utilized full information maximum likelihood estimation to account for missing data [57], and report the Yuan-Bentler rescaled χ^2^ for use with robust estimation [58]. We evaluated the model in terms of the significance of the loadings and hypothesized structural paths through the use of overall fit measures [59]. Guidelines for cut-off points suggest that values close to or above 0.90 for the comparative fit index (CFI) and 0.07 or lower for the root mean square error approximation (RMSEA) are indicative of good fit. With this criteria, this model was an adequate fit to the data, Yuan-Bentler scaled χ^2^ (41, *n*=172) = 62.97, *p*<0.05, CFI=0.95, RMSEA=0.07. All indicators had significant loadings on their hypothesized latent variables, and the structural paths were significant. Standardized parameter estimates and significance values can be seen in Figure 2. The model was run including gender as a covariate and the size and significance of the paths, as well as interpretations of the outcomes remained unchanged.

The findings indicate that higher genetic risk scores were associated with less activation on the BART and, consistent with prior work [24], higher activation on the BART was associated with less risk behavior. There was the expected positive association between peer norms and risk behavior, though importantly the relationship of neurocognitive activation to behavior was still significant with norms in the model. As is standard practice, the correlation between genetic risk and peer norms was included in the model, but was not significant so is not shown in Figure 2.

## Discussion

Our goal was to demonstrate how neurocognitive and genetic variables can be integrated into a translational framework to better understand alcohol-related sexual risk among juvenile-justice involved youth. The identification of important correlates of alcohol-related sexual risk suggests that, in addition to psychosocial factors, there may be neurocognitive and genetic variables that underlie risk behavior generally, and could potentially moderate the effectiveness of interventions to decrease alcohol-related sexual risk. Developing more effective interventions is one component to reducing the incidence of HIV/STI in youth.

An exploratory structural equation model identified latent variables characterizing one conceptualization of a pathway from genetic risk to neurocognitive activation, and then to alcohol-related risky sexual behavior. As predicted, there were significant associations between genetic risk (the linear combination of CHRM2, CNR1, and DRD4) and neurocognitive activation during the BART. Further, activation during the BART was associated with risk behavior in this sample in ways consistent to its relationship with risk behavior in other samples [24]. These relationships were significant even though an important motivational variable for adolescents, perception of peer risk behavior, was included in the model. This finding suggests that underlying biological substrates may account for unique variability in risk behavior, and support the proposition that such variables might moderate the effectiveness of interventions to change risk behavior.

More broadly, there is rapidly expanding interest in the incorporation of biological variables into prevention research. Beauchaine et al. [60] note that the great strength of incorporating biology in this context is the ability to understand when biology and the environment (here, a behavior change intervention) interact to predict outcomes. The notion that biology moderates the effectiveness of pharmacological interventions has long been recognized and is routinely incorporated into clinical medical practice (e.g., [61]). Biological factors (e.g., genetics, neurocognition) may also moderate response to *behavioral* interventions (c.f., [62]). Further, in the domain of genetic variation, linkages between genetics and neurocognition are crucial, as they provide a mechanistic explanation of why genetics would moderate responsiveness to behavioral interventions. These interventions are, fundamentally, hypothesized to work via changes in cognitive processes (c.f.,[63]). Work like ours is important as it lays the foundational preliminary data to suggest potential biological markers and relationships between them. Because of their relationship with the behavior at focus, these markers may also moderate response to interventions to change that behavior.

Studying these questions in the domain of adolescent risk taking has great potential not only to indicate biological variables that moderate treatment outcome, but to uncover the answers to more basic questions about neurodevelopment. This is due to the fact that cognitive and emotional functioning are undergoing dramatic changes throughout adolescence as a result of normal developmental changes occurring in the brain as well as the increasing influence of peers [64,65]. Measuring neurocognitive function may provide a unique window into the constructs that are important for predicting risk-taking outcomes among adolescents. For example, whereas self-report questionnaires may identify some aspects of impulsivity and/or sensation-seeking, the addition of tasks such as the BART or delay discounting allow for actual decision-making behavior that contributes to the latent constructs of impulsivity/sensation-seeking that ultimately predict future risk taking behavior (e.g. [66,67]).

There were a number of limitations of the current investigation. First, the current sample was derived from a subset of high-risk adolescents and the findings may not generalize to adolescent behavior as a whole. Second, although the total sample was large, correcting for movement and scanning eligibility restrictions reduced the sample. Third, while single-session interventions have been found to significantly influence behavior change in the domain of condom use, Johnson and colleagues [35] did note that multi-session approaches may be important if the goal is to reduce sexual frequency. In an optimal situation, a multi-session intervention might be preferable. However, given the highly constrained logistics of conducting HIV prevention interventions with juvenile-justice youth (e.g., youth are only in programs for a brief time, ease of program staff to implement program, etc.) it may not be feasible to conduct multi-session programs in this context. Finally, the structural equation model was of an exploratory nature based on cross-sectional and retrospective data, and there are other equivalent models that would also adequately represent the relationships in the data. These findings cannot determine causal relationships. Further, these findings not only require replication in general, but also replication in different populations.

Despite the exploratory and cross-sectional nature of the current investigation, the outcomes do provide support for a biopsychosocial approach to HIV/AIDS reduction strategies among juvenile justice involved youth. Specifically, moving beyond psychosocial factors and integrating neurocognitive and genetic factors within such intervention programs may better determine for whom these interventions will be most effective.

## Supplementary Material

Suppl Table 1

## Figures and Tables

**Table 1:** Content of GPI+GMET Intervention.

Section	Duration (mins)	Content
1	10	HIV/STI transmission, behaviors that put people at risk for HIV/STIs, identification of local HIV/STI resources.
2	40	Condom-use self-efficacy, discussion of obtaining, using, storing, and discussing condoms.
3	25	Discussion of consequences, attitudes and norms about condom use, vicarious role-play using video.
4	60	GMET, discussion of safer sex and alcohol reduction strategies
5	10	Setting future goals for safer sexual behavior
**Total**	**145 minutes**	

**Table 2:** Baseline Characteristics (N=284).

	Female (N=79)	Male (N=205)	Total (N=284)
**Demographics**			
Ethnicity (%Hispanic)	64.6%	62.4%	63.0%
Age	16.13 (1.14)	16.17 (1.08)	16.16 (1.09)
**Individual Differences**			
Impulsivity(0–19)	10.95 (4.18)	10.55 (3.82)	10.67 (3.92)
Externalizing (0–64)	20.56 (11.22)	21.90 (12.95)	21.52 (12.48)
Depression(0–20)*	3.96 (3.44)	2.65 (3.09)	3.03 (3.24)
AD/HD symptomology (0–81)	27.91 (12.98)	29.22 (14.93)	28.85 (14.40)
**Sexual Behavior**			
% LT sexually active	78.5%	80.7%	80.1%
First intercourse age*	14.00 (1.69)	12.79 (2.07)	13.12 (2.04)
LT sexual partners*	4.94 (6.48)	6.77 (6.48)	6.24 (6.52)
LT frequency of intercourse (1–6)	3.08 (1.39)	3.02 (1.48)	3.04 (1.45)
LT condom use (1–5)	3.13 (1.18)	3.35 (1.18)	3.29 (1.18)
LT alcohol during intercourse (1–5)	2.05 (0.96)	2.31 (0.96)	2.24 (0.97)
% LT positive STI*	21.0%	6.2%	10.3%
% LT been/gotten someone pregnant	22.6%	22.7%	22.7%
3Mfrequency of intercourse (1–6)	2.64 (1.63)	2.74 (1.57)	2.72 (1.58)
3M condom use (1–5)	2.86 (1.38)	3.18 (1.35)	3.09 (1.36)
3M alcohol during intercourse (1–5)	1.83 (0.99)	2.02 (1.00)	1.97 (1.00)
30D sexual partners*	1.18 (0.68)	1.77 (1.76)	1.63 (1.59)
30D intercourse days	3.34 (6.32)	4.31 (6.40)	4.04 (6.38)
30D avg % intercourse days with condom	35.6 (48.2)	50.5 (45.9)	47.1 (46.7)
30D alcohol and intercourse days	1.15 (2.78)	1.73 (2.83)	1.60 (2.82)
**Alcohol Use**			
% LT used alcohol	91.0%	90.3%	90.5%
AUDIT	7.42 (8.07)	8.08 (6.98)	7.90 (7.29)
RAPI	13.28 (15.30)	13.20 (15.55)	13.22 (15.45)
First drink age*	13.12 (2.27)	12.35 (2.25)	12.57 (2.28)
3M alcohol use composite	8.28 (5.21)	8.84 (4.79)	8.68 (4.91)
30D drinking days	2.51 (4.60)	3.45 (4.81)	3.18 (4.76)
30D drinks/drinking day*	3.72 (4.95)	5.25 (6.09)	4.82 (5.83)
30D binge drinking days	2.25 (4.58)	2.58 (4.14)	2.49 (4.26)
Peer Norms	9.94 (2.28)	9.73 (2.35)	9.79 (2.33)
**Motivation for Safer Sexual Behavior**			
Intentions	2.76 (0.69)	2.77 (0.66)	2.77 (0.67)
Attitudes *	3.11 (0.49)	2.86 (0.50)	2.93 (0.51)
Global attitudes	5.42 (1.18)	5.19 (1.36)	5.25 (1.31)
Self-efficacy	3.47 (0.44)	3.45 (0.43)	3.46 (0.43)
Peer norms	2.65 (0.88)	2.79 (0.84)	2.75 (0.85)

*Note.* LT=lifetime, 3M=three months, 30D=30 days.

* Significant gender difference, *p*<.05. Values represent mean and standard deviation unless a percentage is provided.
