# Supplementary material for: Evaluating an Integrative Theoretical Framework for HIV Sexual Risk among Juvenile Justice involved Adolescents
Source: J AIDS Clin Res. Author manuscript; Available in PMC 2014 Aug 11. (PMC4128495)
Supplement: Suppl Table 1 [file NIHMS591756-supplement-Suppl_Table_1.docx]

Supplemental Table 1.

Correlations between recent sexual behavior, alcohol use, and motivation for safer sexual behavior.

|  | 1 | 2 | 3 | 4 | 5 | 6 | 7 | 8 |
| --- | --- | --- | --- | --- | --- | --- | --- | --- |
| 1. freq of intercourse (3M) | **--** |  |  |  |  |  |  |  |
| 2. freq of condom use (3M) | -.06 | **--** |  |  |  |  |  |  |
| 3. Alcohol during intercourse (3M) | **.14*** | .01 | -- |  |  |  |  |  |
| 4. # sexual partners (30D) | .09 | .03 | **.17*** | **--** |  |  |  |  |
| 5. # intercourse days (30D) | **.45***** | **-.15*** | .04 | **.23**** | **--** |  |  |  |
| 6. % intercourse days with condom (30D) | -.08 | **.61***** | .03 | .12 | **-.18*** | -- |  |  |
| 7. # drinking and intercourse days | **.21**** | **-.20*** | **.32***** | **.38***** | **.46***** | -.12 | **--** |  |
| 8. Alcohol use self-report composite (3M) | **.22***** | -.02 | **.37***** | -.06 | **.10 ^a^** | -.05 | **.37***** | **--** |
| 9. # drinking days (30D) | **.16**** | -.10 | **.38***** | **.15^a^** | **.19**** | -.12 | **.69***** | **.46***** |
| 10. # drinks/ drinking day (30D) | **.14*** | -.06 | **.30***** | .06 | .09 | -.03 | **.36***** | **.46***** |
| 11. # binge days (30D) | **.15**** | -.08 | **.37***** | .11 | **.15**** | -.09 | **.59***** | **.43***** |
| 12. Alcohol use peer norms | .04 | -.04 | **.14*** | -.07 | .09 | -.10 | **.15*** | **.35***** |
| 13. CU attitudes | **-.15*** | **.41***** | -.07 | -.04 | **-.17**** | **.32***** | **-.19*** | **-.12^a^** |
| 14. CU global Attitudes | **-.17**** | **.37***** | -.06 | -.01 | **-.25***** | **.41***** | **-.24**** | -.05 |
| 15. CU norms | **-.22***** | **.35***** | .04 | .00 | **-.13*** | **.24***** | -.02 | .02 |
| 16. CU self-efficacy | .04 | **.25***** | -.10 | **.13^a^** | -.04 | **.25***** | -.09 | .03 |
| 17. CU intentions | **-.11^a^** | **.47***** | -.02 | .07 | **-.16**** | **.41***** | -.12 | -.07 |

*Note.* CU = condom use. Bolded items are significant relationships of *p*<.10 or less. ^a^denotes *p<*.10, *denotes *p*<.05, **denotes *p*<.01, ***denotes *p*<.001.

|  | 9 | 10 | 11 | 12 | 13 | 14 | 15 | 16 | 17 |
| --- | --- | --- | --- | --- | --- | --- | --- | --- | --- |
| 1. freq of intercourse (3M) |  |  |  |  |  |  |  |  |  |
| 2. freq of condom use (3M) |  |  |  |  |  |  |  |  |  |
| 3. Alcohol during intercourse (3M) |  |  |  |  |  |  |  |  |  |
| 4. # sexual partners (30D) |  |  |  |  |  |  |  |  |  |
| 5. # intercourse days (30D) |  |  |  |  |  |  |  |  |  |
| 6. % intercourse days with condom (30D) |  |  |  |  |  |  |  |  |  |
| 7. # drinking and intercourse days |  |  |  |  |  |  |  |  |  |
| 8. Alcohol use self-report composite (3M) |  |  |  |  |  |  |  |  |  |
| 9. # drinking days (30D) | -- |  |  |  |  |  |  |  |  |
| 10. # drinks/ drinking day (30D) | **.51***** | -- |  |  |  |  |  |  |  |
| 11. # binge days (30D) | **.93***** | **.55***** | **--** |  |  |  |  |  |  |
| 12. Alcohol use peer norms | **.24***** | **.21***** | **.24***** | **--** |  |  |  |  |  |
| 13. CU attitudes | **-.14*** | -.06 | **-.12*** | **-.13*** | -- |  |  |  |  |
| 14. CU global Attitudes | **-.16**** | -.07 | **-.12 ^a^** | -.02 | **.53***** | -- |  |  |  |
| 15. CU norms | -.01 | .05 | .02 | .00 | **.23***** | **.26***** | **--** |  |  |
| 16. CU self-efficacy | -.04 | .02 | -.03 | -.01 | **.50***** | **.42***** | **.29***** | -- |  |
| 17. CU intentions | -.05 | .03 | -.04 | -.06 | **.45***** | **.51***** | **.42***** | **.42***** | **--** |

Table 3 (continued)
